# How can artificial intelligence decrease cognitive and work burden for front line practitioners?

**DOI:** 10.1093/jamiaopen/ooad079

**Published:** 2023-08-29

**Authors:** Tejal K Gandhi, David Classen, Christine A Sinsky, David C Rhew, Nikki Vande Garde, Andrew Roberts, Frank Federico

**Affiliations:** Press Ganey Associates LLC, Boston, MA 02109, United States; Division of Epidemiology, University of Utah School of Medicine, Salt Lake City, UT 84132, United States; Professional Satisfaction & Practice Sustainability, American Medical Association, Chicago, IL 60611, United States; Worldwide Commercial, Microsoft, San Francisco, CA 94103, United States; Patient Safety, Oracle Health, Kansas City, MO 64138, United States; Data Science, Oracle Health, Kansas City, MO 64138, United States; Institute for Healthcare Improvement, Boston, MA 02109, United States

**Keywords:** artificial intelligence, safety, cognitive burden, work burden, front line practitioners

## Abstract

Artificial intelligence (AI) has tremendous potential to improve the cognitive and work burden of clinicians across a range of clinical activities, which could lead to reduced burnout and better clinical care. The recent explosion of generative AI nicely illustrates this potential. Developers and organizations deploying AI have a responsibility to ensure AI is designed and implemented with end-user input, has mechanisms to identify and potentially reduce bias, and that the impact on cognitive and work burden is measured, monitored, and improved. This article focuses specifically on the role AI can play in reducing cognitive and work burden, outlines the critical issues associated with the use of AI, and serves as a call to action for vendors and users to work together to develop functionality that addresses these challenges.

## Introduction

To deliver safe care, physicians and other healthcare workers need to quickly develop an understanding of the patient, their context (ie, medical and social history) and the details of their current care. The information and technological environment in which care is delivered can either facilitate or impede the rapid acquisition of this information. The vast array of electronic data and increasing amounts of that data have resulted in overwhelming and often conflicting data that clinicians must synthesize to make optimal care decisions.

Artificial intelligence (AI) is being aggressively and rapidly developed and implemented across US healthcare. Electronic health record (EHR) vendors are now building AI into their products, as are other vendors such as clinical data warehouse vendors and large technology companies. Often, AI deployment is focused on cost savings, reducing complications, improving care quality, or drug development. Recently, the ONC has published new detailed guidance on AI and decision support interventions to improve transparency, promote trustworthiness, and incentivize the development and wider use of fair, appropriate, valid, effective, and safe decision support for patients.^1^ However, minimal AI development or deployment has been focused on its impact on workload or cognitive burden for frontline workers,^2^^,^^3^ an issue that has become a primary concern due to severe staffing shortages among all types of healthcare workers. This article reviews critical issues for the use of AI to reduce cognitive and work burden for frontline healthcare workers.

## Will AI hinder or help?

As with all people, clinicians have a limited mental bandwidth with which they can meet any situation. Cognitive load is the load imposed on our working memory by a particular task. When cognitively overloaded, our brain processing slows, we incur attentional blinks or blind spots, and we make more errors. Cognitive load theory identifies limitations in working memory that humans depend on to perform cognitive tasks.^4^ Cognitive load is the amount of working memory used and is determined by:

- How intrinsically difficult the task is (intrinsic load);
- How skilled we are at the task; the more skilled we are, the less drain on our working memory (ie, the less the germane load);
- How much work it takes to obtain and process critical pieces of information needed to complete the tasks (ie, extraneous load, often impacted by environmental and systems factors as well as the way information is presented).

Understanding these types of loads as well as the impact on working memory is essential,^5^ in particular because higher cognitive load is associated with increased levels of clinician burnout.^6^^,^^7^ When effectively designed and implemented, AI has the potential to reduce the clinician’s cognitive burden. For this discussion, AI includes the broader group of capabilities in which computer systems are developed to mimic human behaviors or enhance human capabilities such as visual perception, speech recognition, decision-making, and translation between languages. Machine learning is a subset of AI that uses statistical techniques to identify patterns in the data and create new insights and workflow optimizations.

AI, when done well, usually falls into 1 of 2 categories: (1) Makes it easier to do what you already want to do (or even does it for you); or (2) Disrupts your process when you are about to do something you don’t want to do or shouldn’t do (eg, alerting you when you are about to administer a medication to a patient who is allergic). When combined with well-designed and implemented technology, AI has the potential to reduce the extraneous load, making it easier to quickly review and assimilate critical information needed to perform tasks required for safe patient care.

Alternatively, AI and technology that fragments information, creates distractions, or otherwise impedes the synthesis of critical knowledge will add to extraneous load, and increase the likelihood that the healthcare worker will make unsafe clinical decisions. This has been the challenge of clinical decision support, which has been associated with alert fatigue and other workflow disruptions. AI tools must be well-implemented and monitored for unintended consequences to optimize their benefits.

## AI’s potential to decrease clinician cognitive and work burden

Decreasing cognitive workload and the work burden that our front-line healthcare workers experience on a daily basis—while maintaining or improving quality and safety of care—requires improving time-consuming clinical processes that are often manually performed by busy healthcare workers.

There are many case examples that delineate the value of AI in facilitating clinicians’ cognitive functions and clinical processes, such as data gathering, synthesis, documentation, and taking appropriate action (Table 1). For example, advances in natural language processing have enabled the ability to parse out key data elements from unstructured text; this is a major contributor to the data gathering and synthesis process and can be leveraged in applications to improve clinicians’ workflows. The recent release of Chat GPT 4 and the other large language models like Microsoft’s Bing chatbot shows AI can play a role in reducing burden on frontline providers.^8^ Indeed, a major EHR vendor just announced the incorporation of Chat GPT 4 into its EHR product to help reduce work burden on frontline clinicians.^9^

**Table 1.** Clinical and cognitive processes potentially impacted by AI.

Function	Description	Example
Data gathering.	AI-based tools such as natural language processing (NLP), large language models, and image recognition can help clinicians comb through large volumes of information sources and discrete or unstructured data to help locate, identify, and surface the most relevant pieces of information, as well as missing information, to reduce cognitive and work burden on clinicians.	*Data gathering*: Using NLP, clinicians can more efficiently search genomic and clinical trial databases, medical literature, and other sources of information to rapidly identify recommended treatments specific to cancer patients.
		*Data gathering and synthesis*: Solutions identify and compile both structured and unstructured EHR data and present that information to the provider to identify potential missing diagnoses, diagnoses that lack specificity, and documented diagnoses that lack clinical evidence. Presenting this information directly to providers within their workflow eliminates the need to address manual queries hours, days, or weeks later; thus, reducing the cognitive burden on the provider.
Data synthesis. rapidly collect, organize, manage, and making sense of the datasets from clinical assessments, physiologic observations, and documentation.	AI may be used to help support a new clinical workflow (eg, command center; rapid response team) or to improve an existing one (eg, prioritize existing care management outreach based on risk)	*Data gathering and synthesis*: The future of healthcare includes the use of wearable devices and remote monitoring which will present clinicians with an overwhelming amount of information to sift through to make a clinical decision. AI can help filter through data, identifying critical data points or information that may indicate a change in a patient’s condition or sudden deterioration. AI can also suggest treatments based on scientific evidence, as well as offer customization based on the patient’s condition, ability to adhere to treatment, and personal desires.
Documentation.	AI may be used to create or enter a summary of or provide specific details regarding a patient encounter into an EHR or other system of record using technology. AI can help simplify the billing documentation process, and generative AI can help reduce documentation burden.	*Documentation*: Ambient clinical intelligence (ACI) allows patients and clinicians to engage in conversation without the clinician needing to focus his/her time on a keyboard or screen. When used in ACI, AI helps identify the speaker, uses NLP to convert the voice conversation into text, maps the medical terms and phrases to standardized nomenclatures, and organizes the conversation into a properly formatted clinical note, that can then be reviewed by the clinician for accuracy and relevance. Once finalized, the note is integrated into the EHR. Voice can also be leveraged to navigate and act within the EHR.
Taking appropriate action.	AI can provide decision support, prediction tools, targeted outreach, and guidance in response to signals or deploy an “intelligent” command center to help manage populations	*Data gathering, synthesis and taking action*: In 2018, Ochsner Health System (New Orleans, LA) leveraged a pilot program to redesign care models at several campuses to include virtual nursing.^14^^,^^15^ With trained clinicians working from remote command centers or virtual bunkers, there is opportunity to leverage AI to remove interruptions for nurses and physicians at the patient’s bedside, and to centralize AI and ML model monitoring and management while allowing associated interventions to continue to be carried out at the bedside.
		*Taking action*: Clinical decision support, particularly related to medication prescribing, has been plagued with issues related to over-alerting, contributing to cognitive burden. In a recent study, researchers at one academic medical center found that ML techniques could enable intelligent filtering of medication alerts and reduce alert volume by 54%.^16^
Structure asynchronous activities, outcomes and activities.	The care team’s clinical and operational activities, including patient rounds and communication, are unstructured and asynchronous. AI can help manage coordination and synthesis of the information for updates and the next best course of action.	*Data gathering, synthesis and taking action*: A computer assisted management program for antibiotics and other anti-infective agents showed improved patient and antibiotic outcomes.^17^

## Design and implementation

AI holds the power to rapidly synthesize large datasets from clinical assessments, physiologic observations, and documentation to derive a score faster than the human brain. Much like any other assessment or clinical tool used to assess risk and guide interventions, clinicians must understand the output of such data to inform their plan of care, in combination with other inputs such as real-time clinical assessments and patient preferences. Clinicians must also be provided appropriate product labeling and methodology disclosures by AI tool developers.

To successfully implement AI and machine learning to reduce frontline cognitive and workflow burden requires engaging both internal and external stakeholders. AI tools can be designed by vendors working with health systems, or by health systems themselves. Frontline staff engagement is essential for user-centered design, implementation, and adaptation of the new practice for both implementation success and reduction of cognitive burden, particularly because in healthcare, software developers are not system end users. These frontline stakeholders include clinical care delivery personnel and frontline technical experts in health IT, bioengineering, system redesign, and research. Staff engagement will only succeed if supported by frontline management and organizational leaders.

In addition, successful implementation requires organizations to design for sustainability from the start, empowering local stakeholders to be actively involved in all aspects of planning, delivering, measuring, and refining a practice so they feel ownership of, and understand the value of, the practice. This leads to more success in adapting the practice to their context and identifying the means to sustain a practice once initial funding is expended.

Frontline design must also be supported by key external stakeholders, who in this case are EHR vendors, clinical data warehouse vendors, command center vendors, patient advocates, and researchers. How might these stakeholders work together? A new, novel set of health IT standards may offer such a model. These novel new standards, developed by the Association for the Advancement of Medical Instrumentation (AAMI), are called software life cycle standards because they apply to every stage of software—from development to implementation to ongoing use, maintenance, and ultimately retirement—based on a successful model in aviation software regulation.^10^ These standards outline the critical role of frontline users in software development, as opposed to traditional standards that only focused on software developers’ roles in software design. These standards also focus on the critical role of human factors in software development, implementation, and use, defining and outlining best practices to conduct user-centered design. To ensure frontline involvement at all stages of the software life cycle will require establishing successful collaborative partnerships among internal and key external stakeholders.

Of note, a significant challenge associated with designing and implementing AI is the lack of data standards within the health systems, vendors, and at the national level. Most automated synthesis in healthcare today requires standardization, but that standardization can significantly reduce data fidelity—a challenge that is unavoidable but that not be ignored. For example, categories of nursing units are important data that likely have not been a focus for standardization. Yet in care models, it is important to differentiate a step-down unit from an intensive care unit, or a neonatal intensive care unit from the nursery.

## Monitoring performance related to cognitive burden

Whenever new technologies or treatments are deployed, there is a need to constantly assess for performance and unintended consequences—and when identified, explore the reasons for these events and proactively address the root causes. Known best practices for monitoring AI include scheduled review of AI algorithm content and performance, monitoring data inputs, identifying drift, and assessing key performance indicators.

When implementing AI to reduce clinicians’ cognitive burden, health systems and vendors should measure and monitor key performance indicators. Reliable and accessible measures of cognitive load and workload are necessary to understand baseline state and to determine whether AI-assisted interventions have resulted in improvement (Table 2). Non-obtrusive metrics, when possible, are preferable to surveys and self-recordings of time, to avoid additional burden on health care professionals. Fortunately, multiple non-invasive workload measures are currently available, such as EHR event log data and time-motion observations.^11^^,^^12^ In addition, cognitive load should be measured at multiple points in the development life-cycle (project inception, pre-model development, pre-deployment, deployment).^3^

**Table 2.** Measures of cognitive load and workload.

EHR event log measures	EHR event log data can quantify the total time a clinician spends within the EHR, as well as time spent on individual tasks—such as documentation and inbox.^11^
	Event log measures detailing “work outside of work” show clinician time spent within the EHR outside of patient scheduled hours.
End user surveys of cognitive load and technology usability	The NASA Task Load Index measures cognitive load and includes dimensions of mental, physical, and temporal demand, as well as effort, performance and frustration level.^18^
	The System Usability Scale (SUS) has been used to assess technology across multiple sectors, including healthcare.^19^
Direct observation	Time motion analysis^20^
	Embedded measures of eye movements
	Numbers and types of interruptions (hard stop vs interruptive vs passive)
End user surveys of consequences of cognitive and work overload	Employee engagement surveys that measure resilience, decompression, and activation
	Measurement of burnout,^21^ work intentions,^22^ and career regret^23^

Lastly, in order to fully recognize the benefits of AI for cognitive burden, it will be essential to understand the return on investment of AI and avoid knee-jerk reactions to leverage AI to justify having less staff. In some cases, that may be appropriate, while in others, reducing staff will only add additional work to those left behind, and increase their cognitive loads yet again (which will counteract the potential benefits). Organizations will need to build robust measurement and monitoring methods to ensure that they are truly optimizing the impact on their staff, and not just focusing on cost savings.

## Addressing bias

Bias and inequities exist in healthcare. AI has the potential to reinforce, as well as help mitigate, those biases. Bias can originate from the data, from those using the data or developing algorithms, or from inappropriate usage of an algorithm’s results.

AI bias is usually derived from statistical bias in existing data; for instance, biases that already exist—either in sample size or human bias in data collection or actions—become encoded in an algorithm. AI-based algorithms are derived and validated using patient datasets that may have limited size and diversity. This places an even greater importance on active monitoring and surveillance of outcomes after AI-based technologies have been developed, validated, and deployed. Real-world data, leading to real-world evidence, can provide a feedback loop to developers, allowing them to refine and adjust AI algorithms when appropriate.^13^

Another critical issue is AI algorithms often include demographic factors such as age, race, ethnicity, gender, and religion in their risk assessments. However, when used to identify eligibility for essential healthcare products or services in limited supply—such as life-saving medications or surgeries—AI algorithms can worsen health disparities. To address this, it is important to understand the underlying reasons why demographic or lifestyle factors are associated with adverse outcomes, attempt—as much as possible—to remove algorithm biases, and ensure that AI application is appropriate and ethical. Ultimately, it is important to have transparency about what the algorithm includes and publish results and performance metrics. For instance, developers could build multiple models (eg, one with demographics and one without), to better understand the potential presence of bias in the algorithm. As healthcare examines and segments various sources of data to identify bias, it will be necessary to continue to monitor those metrics after AI implementation.

Importantly, AI can also be part of the solution to bias. For example, when inequities exist—differential referral patterns for patients of different racial or ethnic groups for similar symptoms—AI can be designed to reduce those inequities and de-bias the referral patterns. AI has potential to improve the cognitive and work burden of clinicians across a range of clinical activities, which could lead to reduced burnout and better clinical care.

## Conclusion

With recent developments in generative AI and other AI applications in healthcare, we are just at the beginning of the AI revolution and substantial contributions to healthcare are coming. However, the use of AI to reduce cognitive and work burden must be a priority. To take advantage of AI’s full potential and to minimize potential downsides, vendors and users must work together to develop AI functionality that addresses these issues. It is time to develop a pilot version of each of the 3 leading EHR vendors’ software that operationalizes the many advances in reducing cognitive and work burden outlined in this article, and conduct a rigorous evaluation by a well-established research team—with public grant funding—that coordinates cross-site evaluation. The evaluation should include both inpatient and ambulatory use cases. Both research results and functionality content should be published and widely shared. Developers and organizations deploying AI have a responsibility to ensure that it is designed and implemented with end-user input, has mechanisms to reduce and potentially even improve bias, and measures and evaluates AI’s impact on cognitive and work burden.

## Data Availability

No new data were generated or analyzed in support of this research.

## Notes

The opinions expressed in this article are those of the authors and should not be interpreted as representing the views of their employers.

## References

[1] Office of the National Coordinator for Health Information Technology (ONC), Department of Health and Human Services (HHS). Health Data, Technology, and Interoperability: Certification Program Updates, Algorithm Transparency, and Information Sharing. 2023. Accessed July 27, 2023. https://www.federalregister.gov/documents/2023/04/18/2023-07229/health-data-technology-and-interoperability-certification-program-updates-algorithm-transparency.

[2] Classen DC , Longhurst C, Thomas EJ. Bending the patient safety curve: how much can AI help? NPJ Digit Med. 2023;6(1):2. 10.1038/s41746-022-00731-5 36599913 PMC9811862

[3] Ehrmann DE , Gallant SN, Nagaraj S, et al Evaluating and reducing cognitive load should be a priority for machine learning in healthcare. Nat Med. 2022;28(7):1331-1333. 10.1038/s41591-022-01833-z 35641825

[4] Sweller J. Cognitive load theory. In: Mestre JP, Ross BH, eds. The Psychology of Learning and Motivation: Cognition in Education. Vol 55. San Diego: Academic Press; 2011:37-76.

[5] National Aeronautics and Space Administration. Stress, Cognition, and Human Performance: A Literature Review and Conceptual Framework. Staal M. NASA/TM-2004-212824. 2004. Accessed July 31, 2023. https://hsi.arc.nasa.gov/publications/20051028105746_IH-054%20Staal.pdf.

[6] Melnick ER , Harry E, Sinsky CA, et al Perceived electronic health record usability as a predictor of task load and burnout among US physicians: mediation analysis. J Med Internet Res. 2020;22(12):e23382. 10.2196/23382 33289493 PMC7785404

[7] Harry E , Sinsky C, Dyrbye LN, et al Physician task load and the risk of burnout among US physicians in a national survey. Jt Comm J Qual Patient Saf. 2021;47(2):76-85. 33168367 10.1016/j.jcjq.2020.09.011

[8] Ayers JW , Poliak A, Dredze M, et al Comparing physician and artificial intelligence chatbot responses to patient questions posted to a public social media forum. JAMA Intern Med. 2023;183(6):589-596. 10.1001/jamainternmed.2023.1838 37115527 PMC10148230

[9] Diaz N . Epic looks to accelerate generative AI offerings. 2023. Accessed August 23, 2023. https://www.beckershospitalreview.com/ehrs/epic-looks-to-accelerate-generative-ai-offerings.html?utm_campaign=bhr&utm_source=website&utm_content=latestarticles.

[10] Association for the Advancement of Medical Instrumentation. Safety and effectiveness of health IT software and systems—Part 4: Application of human factors engineering. 2020. Accessed August 7, 2023. https://store.aami.org/s/store?_gl=1*7z3ey5*_ga*MTE5NDA5MjQ4Mi4xNjkyODIyMzE4*_ga_PMV3KJP116*MTY5MjgyMjMxNy4xLjEuMTY5MjgyMjMzMi4wLjAuMA..#/store/browse/detail/a152E000006jlKKQAY.

[11] Sinsky CA , Rule A, Cohen G, et al Metrics for assessing physician activity using electronic health record log data. J Am Med Inform Assoc. 2020;27(4):639-643. 10.1093/jamia/ocz223 32027360 PMC7075531

[12] Arndt BG , Beasley JW, Watkinson MD, et al Tethered to the EHR: primary care physician workload assessment using EHR event log data and time-motion observations. Ann Fam Med. 2017;15(5):419-426. 10.1370/afm.2121 28893811 PMC5593724

[13] Proposed regulatory framework for modifications to artificial intelligence/machine learning (AI/ML)-based software as a medical device (SaMD). U.S. Food and Drug Administration; 2019. Accessed February 15, 2023. https://www.fda.gov/media/122535/download.

[14] Schwamm LH , Estrada J, Erskine A, Licurse A. Virtual care: New models of caring for our patients and workforce. Lancet Digit Health. 2020;2(6):E282-E285. 10.1016/s2589-7500(20)30104-7 32382724 PMC7202848

[15] Heyworth L , Kirsh S, Zulman D, Ferguson JM, Kizer KW. Expanding access through virtual care: the VA’s early experience with Covid-19. *NEJM Catalyst*. 2020. Accessed February 15, 2023. https://catalyst.nejm.org/doi/full/10.1056/CAT.20.0327.

[16] Liu S , Kawamoto K, Del Fiol G, et al The potential for leveraging machine learning to filter medication alerts. J Am Med Inform Assoc. 2022;29(5):891-899. 10.1093/jamia/ocab292 34990507 PMC9006688

[17] Evans RS , Pestotnik SL, Classen DC, et al A computer-assisted management program for antibiotics and other antiinfective agents. N Engl J Med. 1998;338(4):232-238. 10.1056/nejm199801223380406 9435330

[18] NASA task load index. NASA Task Load Index. Accessed February 15, 2023. Digital Healthcare Research. https://digital.ahrq.gov/health-it-tools-and-resources/evaluation-resources/workflow-assessment-health-it-toolkit/all-workflow-tools/nasa-task-load-index.

[19] System usability scale (SUS). Usability.gov. 2013. Accessed February 15, 2023. https://www.usability.gov/how-to-and-tools/methods/system-usability-scale.html.

[20] Sinsky C , Colligan L, Li L, et al Allocation of physician time in ambulatory practice: a time and motion study in 4 specialties. Ann Intern Med. 2016;165(11):753-760. 10.7326/m16-0961 27595430

[21] Brady KJ , Ni P, Carlasare L, et al Establishing crosswalks between common measures of burnout in US physicians. J Gen Intern Med. 2022;37(4):777-784. 10.1007/s11606-021-06661-4 33791938 PMC8904666

[22] Sinsky CA , Dyrbye LN, West CP, Satele D, Tutty M, Shanafelt TD. Professional satisfaction and the career plans of US physicians. Mayo Clin Proc. 2017;92(11):1625-1635. 10.1016/j.mayocp.2017.08.017 29101932

[23] Dyrbye LN , Burke SE, Hardeman RR, et al Association of clinical specialty with symptoms of burnout and career choice regret among US resident physicians. JAMA. 2018;320(11):1114-1130. 10.1001/jama.2018.12615 30422299 PMC6233627

